# Regulatory networks of iron sulfur cluster biology in cancer mechanisms and therapeutic perspectives

**DOI:** 10.1016/j.isci.2026.115256

**Published:** 2026-03-10

**Authors:** Shenshen Yao, Hongbo Guan, Jun Chai, Xiaomei Liu

**Affiliations:** 1 Department of Obstetrics and Gynecology, Shengjing Hospital of China Medical University, Shenyang, P.R. China; 2 Department of Anesthesiology, Shengjing Hospital of China Medical University, Shenyang, P.R. China

**Keywords:** cancer, cell biology, functional aspects of cell biology

## Abstract

Iron-sulfur (Fe/S) clusters are essential cofactors required for mitochondrial metabolism, redox regulation, DNA synthesis, and cellular viability. Defects in their biogenesis or function compromise mitochondrial homeostasis, iron balance, and genome stability, alterations frequently observed in cancer. Growing evidence indicates that Fe/S proteins participate in tumor cell proliferation, metabolic adaptation, oxidative stress tolerance, and therapeutic resistance. This review summarizes current knowledge on the mechanisms of Fe/S cluster assembly in distinct cellular compartments, including the mitochondria, cytosol, and nucleus, and outlines their physiological roles in normal and malignant cells. It further discusses the molecular mechanisms by which dysregulation of Fe/S cluster homeostasis contributes to tumorigenesis. In addition, we highlight emerging therapeutic strategies that exploit Fe/S cluster dependencies, including small-molecule approaches, regulated cell death pathways, and nanomedicine-based interventions. Collectively, these insights underscore the relevance of Fe/S cluster biology to cancer pathogenesis and its potential for therapeutic exploitation.

## Graphical abstract

**Figure:**
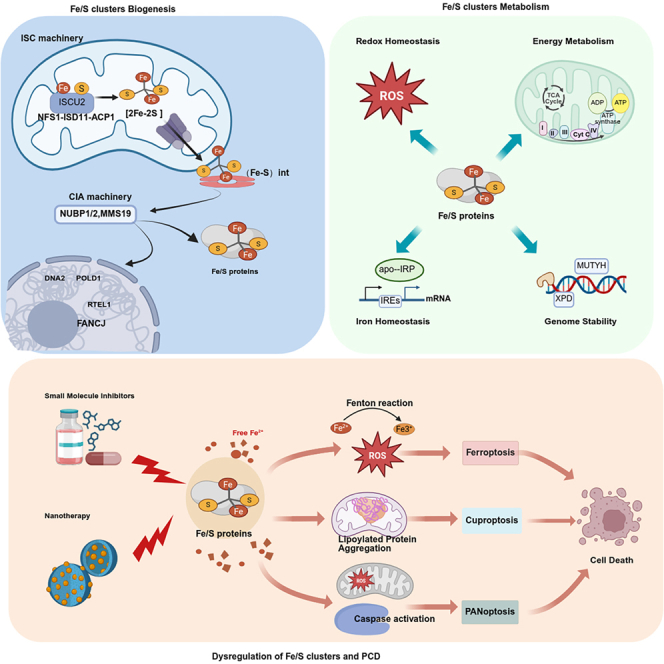

## Introduction

Iron-sulfur (Fe/S) clusters are ancient, versatile cofactors that are universally conserved across all domains of life. These prosthetic groups, which typically exist as [2Fe-2S] and [4Fe-4S], are coordinated by cysteine or histidine residues within proteins. Due to their unique redox and structural properties, Fe/S clusters enable enzymes to participate in essential cellular processes, including mitochondrial energy production, DNA replication and repair, maintenance of iron and sulfur homeostasis, and regulation of programmed cell death (PCD). In mammalian cells, Fe/S clusters are synthesized in mitochondria and transferred to the cytosol and nucleus for incorporation into proteins. Dynamic regulation of Fe/S clusters assembly and trafficking ensures the functional integrity of enzymes that sustain metabolic networks and genomic stability.

Dysregulation of this system leads to mitochondrial dysfunction, iron imbalance, and oxidative stress, which collectively contribute to the pathogenesis of numerous diseases, ranging from Friedreich ataxia (FRDA) and sideroblastic anemia to various forms of cancer. In the oncological context, Fe/S proteins have emerged as critical regulators of tumor metabolism, redox adaptation, and genomic stability. Their dysfunction not only promotes oncogenic transformation and therapeutic resistance but also unveils distinct metabolic vulnerabilities and dependencies that may be exploited for therapeutic intervention.

This review summarizes advances in Fe/S cluster biology, emphasizing biosynthesis, roles in DNA replication and repair, regulation of PCD, and contribution to metabolic reprogramming in cancer research. We highlight emerging therapeutic strategies targeting Fe/S proteins, and discuss the potential of harnessing these pathways for precision oncology in the future.

## Fe/S clusters biogenesis

**Figure 1:**
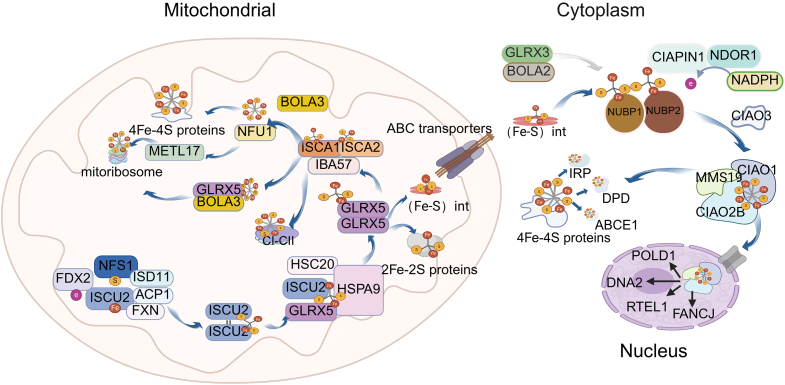
Integrated mechanism of Fe/S protein synthesis in mitochondria, cytosol, and nucleus The assembly of Fe/S clusters is initiated in the mitochondrial matrix by the ISC machinery, where the cysteine desulfurase complex (NFS1-ISD11-ACP1), aided by frataxin (FXN) and the NADH-FDX2 electron transfer chain, synthesizes a [2Fe-2S] cluster on the scaffold protein ISCU2. Following chaperone-mediated release to GLRX5, the cluster is either utilized for mitochondrial [4Fe-4S] proteins or exported as a glutathione-coordinated intermediate, (Fe-S) int, via the IMM transporter ABCB7. In the cytosol, this intermediate is primarily received by the NUBP1-NUBP2 scaffold complex, with the GLRX3-BOLA2 complex serving as an auxiliary chaperone. The maturation of these transient clusters into a stable [4Fe-4S] form on NUBP1-NUBP2 relies on electrons provided by the NADPH-NDOR1-CIAPIN1 cascade. Finally, the cluster is transferred via CIAO3 (IOP1) to the late-acting targeting complex (MMS19-CIAO1-CIAO2B), which specifically inserts Fe/S clusters into cytosolic clients (e.g., IRP and ABCE1) and key nuclear DNA metabolism enzymes (e.g., POLD1, FANCJ, and PRIM1). POLD1, DNA polymerase delta catalytic subunit; DNA2, DNA replication ATP-dependent helicase/nuclease; FANCJ, Fanconi anemia group J protein; RTEL1, regulator of telomere elongation helicase 1; DPD, dihydropyrimidine dehydrogenase. The image was created in https://BioRender.com.

The Fe/S clusters exhibit remarkable chemical diversity. The most common types are [2Fe-2S] and [4Fe-4S] clusters.^1^ In addition, [3Fe-4S] and [8Fe-7S] clusters have been identified.^2^^,^^3^ Under oxidative conditions, [4Fe-4S] clusters can degrade into [3Fe-4S] forms,^4^ whereas the biosynthesis of the more complex [8Fe-7S] cluster requires auxiliary proteins such as NifH and NifZ, as well as ATP hydrolysis and electron transfer.^5^ In eukaryotic cells, the biogenesis of [2Fe-2S] and [4Fe-4S] clusters involve multiple tightly co-ordinated steps, including sulfur mobilization, iron delivery, scaffold assembly, and transfer to recipient apoproteins (Figure 1).

### Mitochondrial assembly of Fe/S clusters

In eukaryotes, the mitochondrial Fe/S clusters assembly (ISC) system synthesizes Fe/S clusters and inserts them into mitochondrial target proteins. The scaffold protein ISCU2 (yeast Isu1 and Isu2) provides a structural platform for Fe/S clusters assembly.^6^ Alternative splicing of *ISCU* gene transcript gives rise to distinct isoforms, among which the mitochondrial-targeted isoform (commonly referred to in the literature as “ISCU2”) plays a central role in mitochondrial ISC. For consistency with previous studies, we use the term ISCU2 throughout this review to denote the mitochondrial isoform of *ISCU* gene product. The scaffold protein ISCU2 associates with the ISC core complex, composed of cysteine desulfurase (NFS1), its stabilizing partner (ISD11, also known as LYRM4), and acyl carrier protein 1 (ACP1), forming a symmetric and catalytically active machinery. In the context of this assembly machinery, ferrous ions bind to the ISCU2 assembly site, which is a prerequisite for sulfur transfer. Subsequently, NFS1 catalyzes the conversion of cysteine to alanine and forms a persulfide intermediate (-SSH) on its conserved active site cysteine residue.^7^ Frataxin (FXN; yeast Yfh1) associates with the Fe-ISCU2-NFS1-ISD11-ACP1 core ISC assembly complex. Although FXN has been shown to bind iron via a negatively charged surface, accumulating structural and biochemical evidence indicates that FXN does not function as a direct iron donor. Instead, FXN acts as a regulatory factor that stimulates sulfur transfer from NFS1 to iron-loaded ISCU2 by inducing conformational changes within the complex, thereby facilitating Fe-S bond formation, while the precise molecular mechanism remains to be fully elucidated.^8^ Mitochondrial FDX2 (yeast Yah1) acts as the electron donor under NADPH-dependent conditions, mediating S^0^ to S^2-^ reduction in ISCU2-SSH for Fe/S clusters synthesis.^9^ In later stages, two ISCU2 monomers, each bound to a distinct ISC core complex, dimerize through transient interactions between N-terminal Tyr35 residues. This dimerization integrates Fe-S units from each ISCU2 monomer, forming a bridged [2Fe-2S] cluster.^10^ Following [2Fe-2S] cluster formation, the ISCU2 dimer dissociates from the core complex and delivers the cluster to Fe/S recipient proteins, ensuring efficient Fe/S protein biogenesis in mitochondria. The iron for Fe/S clusters assembly originates from the mitochondrial labile iron pool, as its chelation inhibits Fe/S clusters biosynthesis.^11^ Mitochondrial ferredoxin provides reducing equivalents to the system. While early studies suggested FDX1 deficiency could impair Fe/S enzyme activity and affect iron distribution,^12^ subsequent research showed FDX2 predominates as the electron donor during Fe/S clusters maturation, as FDX1 loss does not reduce mitochondrial or cytosolic Fe/S protein levels.^13^

The release of [2Fe-2S] clusters from ISCU2 is regulated by molecular chaperones and carrier proteins. In human mitochondria, HSC20 binds to a conserved hydrophobic surface on ISCU2 and targets it to HSPA9. The release of [2Fe-2S] clusters depend on specific chaperones, with HSC20 binding to ISCU2’s conserved hydrophobic region before delivery to HSPA9.^14^ Upon delivery, HSPA9 undergoes ATP-dependent changes that stabilize its interaction with cluster-loaded ISCU2. Although the precise structural effects of HSPA9 binding remain unclear, chaperone engagement modulates ISCU2 conformational dynamics, potentially facilitating cluster transfer.^15^ In mammalian cells, GLRX5 interacts with ISCU2 and mitochondrial chaperone HSPA9. While studies show their interactions, it remains unclear if these components form a stable complex during Fe/S cluster transfer *in vivo* or interact transiently.^16^ GLRX5 homodimers subsequently mediate the delivery of [2Fe-2S] clusters to target apoproteins, thereby completing the maturation of mitochondrial Fe/S proteins. Genetic and biochemical studies in yeast show Grx5 is essential for producing transferable Fe/S-related intermediates needed for cytosolic Fe/S protein biogenesis. Biochemical studies demonstrate mitochondrial Grx5 is crucial for generating transferable Fe-S-related intermediates ((Fe-S) int) for cytosolic Fe/S protein maturation. This intermediate differs from mature mitochondrial [4Fe-4S] cluster, as mitochondria lacking Isa1 or Isa2, defective in [4Fe-4S] assembly, can still support cytosolic iron-sulfur cluster assembly (CIA).^17^

The biosynthesis of [4Fe-4S] clusters in mitochondria is a coordinated process not yet fully understood. The ISCA1-ISCA2 heterodimeric complex forms the core of mitochondrial [4Fe-4S] cluster maturation. The [2Fe-2S] cluster from GLRX5 is delivered to ISCA1-ISCA2 via protein interactions to form [4Fe-4S] clusters.^18^ These [2Fe-2S] clusters undergo reductive coupling to form a [4Fe-4S] cluster, a reaction requiring electrons from mitochondrial ferredoxin FDX2, though the direct electron acceptor remains unknown.^19^ The auxiliary protein IBA57 is crucial for [4Fe-4S] ^2+^ cluster assembly on ISCA1-ISCA2, possibly by regulating redox and structural properties through ISCA2 interaction; however, its precise molecular function remains unclear.^20^ Once assembly is complete, the [4Fe-4S] cluster can be transferred directly from ISCA1-ISCA2 to certain recipient apoproteins (such as mitochondrial aconitase) without the involvement of additional targeting factors.^21^ The cluster can be selectively inserted into specific client proteins, such as respiratory chain complexes I and II and lipoyl synthase (LIAS), via specialized carrier proteins such as NFU1 or IND1.^22^ Under oxidative or stress-related conditions, an alternative pathway involving the [2Fe-2S]-bridged BOLA3-GLRX5 complex may support [4Fe-4S] cluster assembly on NFU1, providing additional flexibility to the mitochondrial Fe-S assembly network. The supply of Fe/S clusters to mitochondrial ribosomes involves two pathways: structural [2Fe-2S] clusters via GLRX5-BOLA3, and [4Fe-4S] clusters for METTL17 via ISCA1-NFU1. METTL17 maturation is essential for synthesizing mtDNA-encoded proteins.^23^

### Export of the (Fe-S) int from mitochondria to the cytosol

In mammalian cells, the cytoplasm and nucleus have independent synthesis mechanisms (CIA) for Fe/S clusters. CIA machinery depends on an (Fe-S) int (termed X–S in yeast), produced in mitochondria and exported via ABCB7 transporter. This intermediate regulates Fe/S cluster synthesis in the cytoplasm and nucleus.^24^

Genetic studies in baker’s yeast (Saccharomyces cerevisiae) show mitochondria supply the cytosol with (Fe-S) int for cytosolic Fe/S protein maturation. Loss of mitochondrial ABC transporter Atm1 (yeast homolog of mammalian ABCB7) causes selective loss of cytosolic Fe/S protein activity, while mitochondrial Fe/S proteins remain unaffected. *In vivo* ^55^Fe radiolabeling experiments showed without Atm1, Fe/S clusters cannot be assembled into cytosolic target proteins.^25^ Lindahl et al. found that Atm1-deficient cells under anaerobic conditions showed no mitochondrial iron accumulation and normal aconitase activity, confirming Atm1 loss impairs cytosolic Fe/S assembly while preserving mitochondrial Fe/S assembly.^26^ Studies showed Atm1 as a key mitochondrial export factor for cytosolic Fe/S proteins. Researchers proposed mitochondria synthesize and export (Fe-S) int for CIA.^27^

In mammalian cells, ABCB7 performs conserved functions. Radiolabeling experiments show cytosolic Fe/S protein maturation depends on mitochondrial ISC activity, ABCB7, and Glutathione (GSH), but not on CIA components and cGrxs early on, suggesting mitochondria supply the cytosol with Fe/S clusters.^28^ However, alternative explanations have been proposed. Some studies have indicated that ABCB7 deficiency initially disrupts the homeostasis of mitochondrial Fe/S proteins and, through the activation of iron regulatory protein (IRP) and impaired iron utilization, subsequently leads to defects in cytosolic Fe/S proteins. These observations suggest that at least part of the cytosolic phenotype may be indirectly caused by mitochondrial dysfunction.^29^ ABCB8, a mitochondrial inner membrane ABC transporter, plays a role in iron homeostasis and cytosolic Fe/S protein maturation. ABCB8 deletion causes mitochondrial iron accumulation and reduces cytosolic Fe/S protein activity, while minimally affecting mitochondrial Fe-S proteins and heme biosynthesis. ABCB8 likely influences cytosolic Fe/S protein maturation by regulating iron homeostasis rather than directly exporting (Fe-S) intermediates, as ABCB7 does.^30^

In mammals, NEET proteins add complexity to this field by acting as [2Fe-2S] cluster carriers, mediating cluster transport across the mitochondrial outer membrane via the MiNT-VDAC1-mitoNEET axis. This model is supported by co-immunoprecipitation, *in vitro* cluster transfer experiments, and computational modeling.^31^ NEET proteins are absent in fungi, and evidence from yeast and human cells shows Atm1/ABCB7-mediated export alone supports CIA. Whether NEET proteins serve as an auxiliary pathway or core component of mitochondrial Fe/S export remains unclear.

Despite these advances, the chemical nature of (Fe-S) int remains unresolved. Studies of Atm1-type transporters have shown their substrate-binding cavities can accommodate glutathione-coordinated metal complexes, and *in vitro* experiments have demonstrated transport of GSH-coordinated [2Fe-2S] clusters.^32^ Consistent with this model, the mitochondrial glutathione transporters SLC25A39/40 are essential for CIA, placing GSH functionally upstream of the Fe/S export step.^33^

### Cytosolic and nuclear Fe/S clusters assembly mechanisms

The CIA system does not synthesize Fe/S clusters *de novo*, but uses (Fe-S) int exported from mitochondria. GLRX3 functions as a homodimeric [2Fe-2S] cluster chaperone upstream of the scaffold complex. Its central (GrxA) and C-terminal (GrxB) glutaredoxin domains operate cooperatively to transfer two [2Fe-2S] clusters to monomeric NUBP1, facilitating their reductive coupling into a [4Fe-4S] cluster. While the N-terminal Trx domain is dispensable for cluster transfer to NUBP1, it is essential for the interaction with the co-chaperone CIAPIN1 (anamorsin).^34^^,^^35^ Subsequently, NUBP1 forms a hetero-oligomeric complex with NUBP2 (orthologous to yeast Nbp35-Cfd1), serving as the scaffold for the *de novo* assembly of [4Fe-4S] clusters.^36^^,^^37^ This complex utilizes conserved C-terminal CPxC motifs to coordinate a surface-exposed, bridging [4Fe-4S] cluster; the presence of NUBP2 confers kinetic lability to this cluster, a property crucial for its facile transfer to downstream carriers such as IOP1 (CIAO3).^38^ Furthermore, as P loop NTPases, the binding and hydrolysis of ATP by the NUBP1-NUBP2 complex drive conformational changes and allosteric regulation that coordinate the dynamic assembly and release of the iron-sulfur clusters Assembly and stable cluster formation require reducing equivalents from the mammalian CIAPIN1-NDOR1 module.^39^ This module transfers electrons from Nicotinamide adenine dinucleotide phosphate (NADPH) to the [2Fe-2S] cluster of CIAPIN1 (yeast Dre2) via NDOR1’s flavin cofactor (yeast Tah18), supporting cluster maturation while not affecting initial scaffold assembly.^40^^,^^41^ Late steps of CIA are regulated by the MMS19-CIAO1-CIAO2B (also known as MIP18 or FAM96B) core complex, which delivers mature [4Fe-4S] clusters to specific nuclear/cytoplasmic client proteins.^42^^,^^43^ MMS19 serves as the central platform for recruiting Fe/S client proteins, while CIAO2B acts as an adapter linking MMS19 and CIAO1, stabilizing the complex and facilitating client protein binding. CIAO3 (also known as IOP1 or NARFL) functions as a crucial cluster carrier bridging the upstream NUBP1-NUBP2 scaffold and the downstream MMS19-CIAO1-CIAO2B targeting complex.^44^ Although earlier RNAi studies based on protein stability proposed CIAO3 as an external factor,^45^ recent proteomic analyses confirm it physically links these sub-complexes to form a dynamic “CIA metabolon.”^46^ Mechanistically, CIAO3 receives the [4Fe-4S] cluster from the scaffold and transfers it to the targeting complex. This role is evolutionarily conserved, as the yeast homolog Nar1 mediates cluster transfer between the Cfd1-Nbp35 scaffold and Cia1. Furthermore, CIAO3’s C-terminal cluster stabilizes its structure, while the N-terminal cluster likely senses cellular iron levels. Some studies also suggest that the CIA2OB-CIAO1-MMS19 complex binds and promotes assembly of most cytosolic and nuclear Fe/S proteins.^47^ The maturation of nuclear iron-sulfur proteins, such as DNA polymerases and helicases, primarily relies on the late-acting CIA targeting complex (MMS19-CIAO1-CIA2B), which recognizes substrates either directly via specific C-terminal motifs or indirectly through adaptor proteins such as PRIM1.^48^^,^^49^ Contrary to the traditional view of exclusive cytoplasmic assembly, recent evidence supports an “*in situ*” maturation model where CIA components localize to specific functional sites, such as the cell nucleus or mitotic spindle, to facilitate cluster insertion, potentially utilizing a nuclear pool of GLRX3-BOLA2.^50^

### Identification and characterization of Fe/S proteins

Fe/S proteins are typically coordinated by cysteine residues, although other residues or substrates may also be involved. The diversity of coordination modes and the oxygen sensitivity of the clusters increase the difficulty in identifying new Fe/S proteins. Traditional methods, such as ultraviolet-visible (UV-vis), electron paramagnetic resonance (EPR), Mössbauer, Nuclear Magnetic Resonance (NMR), and Inductively Coupled Plasma (ICP) analyses, can be used to determine the cluster type, coordination, and oxidation state; however, these approaches have limited throughput and depend on correct cluster assembly. Proteomic strategies, such as isoTOP-ABPP, can identify potential Fe/S proteins by detecting changes in cysteine reactivity. In combination with spectroscopic and elemental analyses, these strategies can be used for the validation of the proposed model. Computational methods, especially AlphaFold2, leverage structural prediction and known cluster coordination patterns to aid in the discovery of new cluster-binding sites, which is particularly useful for large proteins or multi-subunit complexes. Integrating spectroscopic, proteomic, functional validation, and computational predictions into a multidimensional strategy can improve the accuracy of Fe/S protein identification and provide reliable candidates for functional studies.^51^ Recently, mass spectrometry-based proteomics strategies have become powerful complementary tools, utilizing radioactive isotope labeling and liquid chromatography inductively coupled plasma-mass spectrometry (LC-ICP-MS), combined with protein-centered approaches, to indirectly infer Fe/S cluster binding through changes in protein properties. Finally, native mass spectrometry-based proteoform analysis integrates both metal- and protein-centered information to define the identity, coordination chemistry, and cellular function of Fe/S proteins.^52^

## Regulatory networks linking Fe/S clusters metabolism to cancer biology

**Figure 2:**
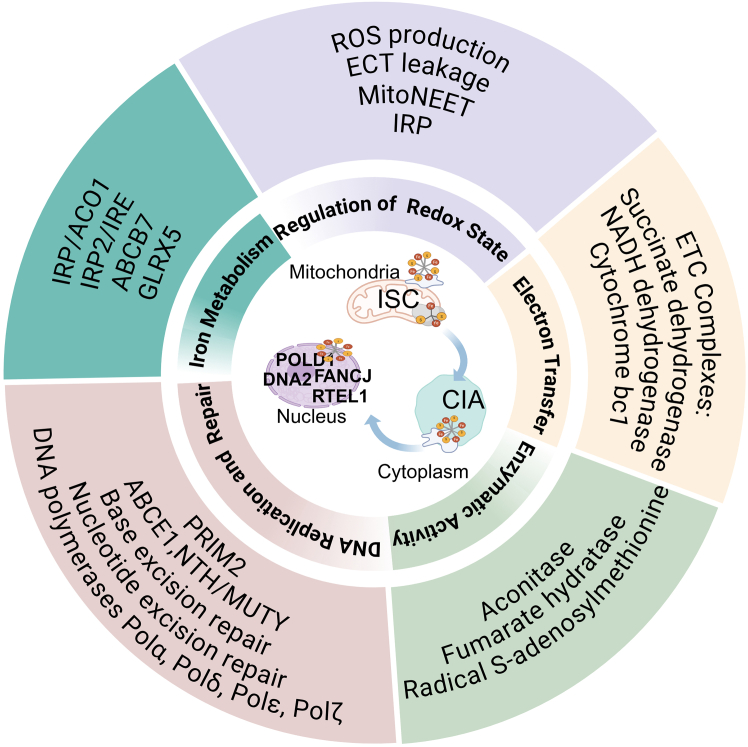
Regulatory networks linking Fe/S clusters metabolism to cancer biology Fe/S clusters act as the central molecular nodes that integrate mitochondrial respiration, redox homeostasis, iron metabolism, and DNA integrity. In cancer cells, alterations in Fe/S clusters assembly or transfer leads to metabolic reprogramming, oxidative stress adaptation, dysregulated iron handling, and impaired genome maintenance, collectively driving tumor survival and progression. The image was created in https://BioRender.com.

Fe/S clusters play multiple essential biological roles in all organisms. Fe/S clusters are indispensable cellular components owing to their remarkable chemical versatility and involvement in numerous key biochemical pathways. Their presence and proper functioning are vital for organisms ranging from simple prokaryotes to complex eukaryotes (Figure 2).

### Electron transfer

Within mitochondria, Fe/S clusters play a crucial role in ATP synthesis and facilitate the function of three primary components of the electron transport chain: Complex I (NADH dehydrogenase), Complex II (succinate dehydrogenase), and Complex III (cytochrome *bc*_1_ complex). These components are instrumental in the transfer of electrons from reduced ubiquinone to cytochrome *c*. This highly organized system enables electrons derived from NADH and FADH_2_ to combine with oxygen, resulting in the formation of water. This process involves the translocation of protons across the inner mitochondrial membrane, resulting in the formation of an electrochemical gradient that facilitates ATP synthase (Complex V) in ATP production.^53^^,^^54^ When Fe/S clusters assemblies malfunction, they impair the efficiency of electron transfer within the respiratory chain, consequently disrupting cellular energy production. NDUFS1, the largest subunit of Complex I, comprises three Fe/S clusters that facilitate electron transfer within the NADH dehydrogenase module and mediate its interaction with Complex III. Biallelic mutations in NDUFS1 destabilize the entire N module and obstruct electron transfer between Fe/S clusters.^55^ NDUFS2 is integral to electron transfer and proton translocation across membranes. The cleft formed by NDUFS2 and NDUFS7 serves as a site for ubiquinone reduction by terminal Fe/S clusters.^56^ Disruption of NDUFS2 significantly impairs cellular growth, Complex I-specific respiration, and ATP production, while concurrently increasing reactive oxygen species (ROS) generation, apoptosis, and necrosis.^57^ Complex II, composed of the SDHA, SDHB, SDHC, and SDHD subunits, transfers electrons from FADH_2_ to ubiquinone via the three 2Fe-2S in SDHB. Mutations in SDHB abolish SDH activity, leading to succinate accumulation and metabolic reprogramming in SDHB-related cancer syndromes.^58^ Disruption of Fe/S clusters impairs mitochondrial oxidative phosphorylation and increases reliance on glycolysis, conferring metabolic plasticity that facilitates tumor survival and metastasis in various cancer types.

### Essential cofactors for enzyme activity

Fe/S clusters function as essential cofactors for multiple enzymes, either by directly engaging with their catalytic centers or by modulating their redox state. In the case of aconitase, its catalytic activity is contingent upon the presence of the [4Fe-4S] cluster, and even the reduced [3Fe-4S] form retains some degree of activity.^59^ The active site of citrate aconitase comprises a [4Fe-4S] cluster that interacts with the hydroxyl group of citrate to facilitate its isomerization.^60^ In yeast, isopropylmalate dehydrogenase (Leu1) functions as a [4Fe-4S] holoenzyme that participates in leucine biosynthesis.^61^ Fumarate hydratase (FH), a tumor suppressor in the Krebs cycle, is frequently mutated in hereditary leiomyomatosis and renal cell carcinoma (HLRCC). Loss of FH activity leads to fumarate accumulation, which succinylates critical cysteine residues in ACO2 required for Fe/S clusters binding, impairs enzymatic activity, and contributes to metabolic dysregulation.^62^ In pancreatic ductal adenocarcinoma (PDAC), ISCU2 expression is significantly upregulated compared to that in the adjacent normal tissues. Oncogenic KRAS enhances c-Myc-mediated ISCU2 transcription, stabilizes Fe/S clusters, and regulates the Tricarboxylic Acid (TCA) cycle enzymes α-ketoglutarate dehydrogenase and ACO2. This promotes both oxidative and reductive TCA cycling and drives α-KG catabolism, leading to reduced cytosolic and nuclear α-KG levels, elevated DNA 5 mC levels, and inhibition of TET3 activity, ultimately enhancing PDAC proliferation and tumor growth.^63^ Additionally, radical S-adenosylmethionine (SAM) enzymes utilize a [4Fe-4S] cluster to generate radical intermediates that catalyze diverse chemical reactions.^64^

### Regulation of cellular redox homeostasis

Fe/S clusters are highly sensitive to redox changes and act as key intracellular redox sensors. For instance, IRP1 binds to iron-responsive elements (IREs) on mRNAs under iron-deficient or oxidative stress conditions, thereby regulating the expression of iron uptake and storage proteins.^65^ The NEET protein family (CISD1-3), comprising [2Fe-2S] cluster-containing mitochondrial proteins, regulates mitochondrial labile iron and ROS levels, and their deletion results in elevated oxidative stress.^66^ Similarly, mitoNEET plays a crucial role in maintaining redox homeostasis and mitigating excessive oxidative stress. Inhibition of mitoNEET has been shown to reduce Lipopolysaccharide (LPS)-induced reactive ROS formation and prevent mitochondrial dysfunction.^67^

### Regulation of iron metabolism

Cellular iron homeostasis is primarily governed by the IRP-IRE system, which coordinates iron uptake, storage, and utilization in response to cellular iron status. IRP function as central post-transcriptional regulators of iron metabolism. Under iron-sufficient conditions, IRP assembles a [4Fe-4S] cluster and adopts aconitase activity, whereas under iron-deficient conditions, Fe/S cluster disassembly converts IRP into an IRE-binding protein, thereby regulating the expression of genes involved in iron metabolism, including transferrin receptor 1 (TfR1) and ferritin.^65^

In cancer cells, iron homeostasis is extensively rewired to support increased proliferative demands, mitochondrial metabolism, and redox balance. IRP, in particular, is frequently stabilized in tumors, leading to enhanced TfR1 expression and suppressed ferritin translation, which collectively expand the labile iron pool and promote tumor growth. Unlike IRP lacks an Fe/S cluster and is primarily regulated at the level of protein stability.

The stability of IRP is controlled by the iron- and oxygen-sensing E3 ubiquitin ligase adaptor FBXL5. FBXL5 contains a C-terminal [2Fe-2S] cluster that is essential for maintaining its structural integrity and enabling recognition of IRP. Under normoxic and iron-replete conditions, the intact Fe/S cluster stabilizes FBXL5, promoting IRP ubiquitination and degradation, thereby preventing iron overload.^68^^,^^69^ Conversely, hypoxia or impaired Fe/S cluster biogenesis destabilizes the FBXL5 Fe/S cluster, leading to IRP accumulation and activation of the iron starvation response—a state frequently exploited by cancer cells to enhance iron acquisition.

Mitochondrial Fe/S cluster biogenesis and export are also critical determinants of cellular iron homeostasis. The mitochondrial ABC transporter Atm1 and its human homolog ABCB7 mediate the export of (Fe-S) int from the mitochondrial matrix to the cytosol, a process essential for CIA. Defects in Atm1 or ABCB7 disrupt CIA, resulting in mitochondrial iron accumulation and compensatory increases in cellular iron uptake. Clinically, ABCB7 mutations are associated with X-linked sideroblastic anemia and ataxia, underscoring the tight coupling between Fe/S cluster trafficking and iron metabolism.^70^

Similarly, the monothiol GLRX5 plays a key role in transferring Fe/S clusters to downstream client proteins, including IRP and ferrochelatase (FECH). GLRX5 deficiency impairs Fe/S cluster delivery, leading to defective IRP regulation, reduced heme synthesis, anemia, and cellular iron overload.^71^ These observations further highlight how perturbations in Fe/S cluster handling can secondarily disrupt iron homeostasis.

Ferritinophagy provides an additional regulatory layer linking iron storage to Fe/S cluster integrity in cancer cells. Nuclear receptor coactivator 4 (NCOA4) functions as a selective cargo receptor that delivers ferritin to lysosomes, thereby releasing stored iron. Recent studies demonstrate that NCOA4 itself acts as an iron- and Fe/S cluster-sensing protein. Under iron-replete conditions, NCOA4 binds Fe/S clusters—reported as [2Fe-2S], [3Fe-4S], or multisite configurations—facilitating HERC2-mediated ubiquitination and limiting ferritinophagy.^72^^,^^73^ In contrast, iron deficiency or impaired Fe/S cluster assembly favors the apo form of NCOA4, enhancing ferritin turnover and increasing intracellular free iron availability.

Collectively, these regulatory mechanisms enable cancer cells to dynamically integrate iron availability with Fe/S cluster biogenesis, mitochondrial function, and redox control. This tight coupling not only supports tumor growth and metabolic adaptation but also critically shapes cancer cell vulnerability to iron-dependent cell death pathways, including ferroptosis.

### Fe/S clusters-dependent regulation of DNA replication and DNA damage repair

In eukaryotic cells, genome duplication depends on B-family DNA polymerases Polα, Polδ, and Polε, along with the translesion polymerase Polζ. Their C-terminal CysB motif coordinates a [4Fe-4S] cluster essential for polymerase stability and assembly. Beyond replication, Fe/S clusters also regulate DNA damage recognition and repair.^74^ In Polδ, Fe/S clusters are ligated by four invariant cysteine residues within the CysB motif. Disruption or loss of this cluster leads to partial enzyme instability, impaired double-stranded DNA binding, and reduced polymerase and exonuclease activities, resulting in replication defects.^75^ Heterozygous missense variants in the exonuclease domain of POLD1 are associated with a cancer susceptibility phenotype.^76^ Similarly, POLE inhibition causes proliferation defects and genomic instability in Basal-Like Breast Cancer (BLBC) cells.^77^ When replicative polymerases encounter replication stress or DNA lesions, error-prone Polζ is recruited to bypass damage. The [4Fe-4S] cluster in Polζ is similarly essential for its function; mutations that disrupt cluster coordination cause severe replication defects and decreased DNA damage tolerance.^78^

The primase subunit PRIM2 also contains a conserved C-terminal [4Fe-4S]-binding domain that facilitates the transition from RNA primer synthesis to DNA elongation during replication initiation. Mutations in cysteine residues that coordinate Fe/S clusters markedly decrease PRIM2 protein stability and result in irreversible S-phase arrest, underscoring the requirement for functional Fe/S clusters for replication initiation.^79^

In addition to replication, Fe/S clusters play an integral role in DNA damage repair pathways through structural and redox-based mechanisms. A particularly intriguing function is DNA-mediated charge transfer (CT). Proteins containing [4Fe-4S] clusters can act as redox sensors and engage in electron transfer through DNA duplexes. Upon DNA binding, the redox potential shifts by approximately −200 mV, positioning the cluster as a physiological redox switch that allows long-range electron signaling between DNA-bound repair proteins.^80^ In the base excision repair (BER) pathway, [4Fe-4S]-containing glycosylases utilize this mechanism to communicate over extended DNA segments, enabling the rapid localization of DNA lesions. When these glycosylases bind to DNA, the oxidation of the [4Fe-4S] ^2+^ cluster to its [4Fe-4S] ^3+^ state generates an electron that travels along the DNA duplex until it encounters another cluster-containing glycosylase, facilitating coordinated lesion detection and repair.^81^

Similarly, the nucleotide excision repair (NER) pathway depends on Fe/S clusters integrity for efficient function. Xeroderma pigmentosum group D (XPD), a component of the TFIIH complex, contains an essential [4Fe-4S] cluster that stabilizes its structure and is required for helicase activity. Mutations or loss of Fe/S clusters assembly disrupt XPD folding, preventing its integration into TFIIH and abolishing helicase activity.^82^ This defect compromises the NER efficiency and increases cellular sensitivity to DNA-damaging agents. Another Fe/S clusters-dependent enzyme, DNA2, functions as a nuclease/helicase for both DNA replication and recombination repair. The absence of Fe/S clusters induces conformational changes that distort the DNA-binding channel, severely impairing substrate interactions and repair capacity.^83^

DNA glycosylases, such as MUTYH, repair small non-helical lesions via the BER pathway, preventing mutagenesis by excising adenines misincorporated opposite oxidized guanines (8-oxoG). The [4Fe-4S] cluster, coordinated by conserved cysteines, stabilizes the enzyme and allosterically links DNA binding to catalytic activity, facilitating efficient lesion recognition and electrostatic interactions with the DNA backbone.^84^^,^^85^ The redox potential of Fe/S clusters is sensitive to DNA structural features, such as abasic sites, influencing DNA-mediated CT that may contribute to damage sensing.^86^ Studies on endonuclease III demonstrate that the [4Fe-4S] cluster’s redox state affects DNA binding and repair activity DNA secondary structures, including G-quadruplex formation or duplex-to-single-stranded switching, modulate CT efficiency and enzyme function,^87^^,^^88^ while spin-dependent CT in chiral DNA assemblies provides additional layers of electronic regulation.^89^ Most mechanistic insights are derived from *in vitro* experiments, and whether long-range electron transfer occurs in living cells remains unclear.

Pathogenic MUTYH variants, including biallelic and somatic loss-of-heterozygosity alterations, underlie colorectal cancer (CRC) in MUTYH-associated polyposis (MAP).^90^ Beyond MAP, these variants increase susceptibility to other tumors, giving rise to the concept of a “MUTYH-associated tumor syndrome.”^91^ Clinical studies show partial or complete loss of MUTYH protein in tumors regardless of genotype, indicating that dysregulation can contribute to tumorigenesis beyond MAP.^92^ Collectively, these findings underscore the critical role of Fe/S clusters in linking MUTYH structure, DNA damage recognition, and repair fidelity.

These regulatory axes collectively define how cancer cells exploit or remodel Fe/S clusters metabolism to sustain growth, resist oxidative stress, and maintain their genomic integrity.

## Dysregulation of Fe/S clusters biology and PCD in cancer

**Figure 3:**
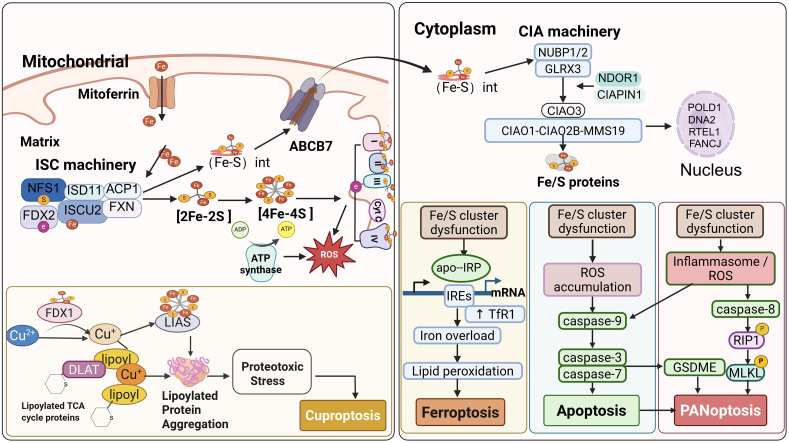
Dysregulation of Fe/S clusters biology and PCD in cancer The diagram illustrates the mitochondrial ISC and cytosolic CIA machineries responsible for Fe/S cluster biogenesis and distribution (via ABCB7). Dysfunctional cluster synthesis links mitochondrial metabolism to three distinct PCD modes: (1) Ferroptosis, driven by apo-IRP activation and subsequent iron overload; (2) Cuproptosis, caused by FDX1/LIAS impairment and protein aggregation; and (3) Apoptosis and PANoptosis, triggered by ROS accumulation and the activation of caspase cascades. The image was created in https://BioRender.com.

Fe/S clusters metabolism is intricately integrated into the regulatory networks that sustain cancer cell homeostasis. When the assembly, trafficking, or maintenance of Fe/S clusters is impaired, a cascade of metabolic and redox imbalances ensues, leading to mitochondrial dysfunction, disrupted electron transfer, and abnormal ROS accumulation in the cell. These disturbances ultimately shift cellular fate from survival to various forms of PCD (Figure 3).

### Fe/S clusters deficiency and ferroptosis

Ferroptosis is a regulated form of cell death driven by iron-dependent lipid peroxidation and disruption of redox homeostasis. Excess intracellular iron catalyzes ROS generation via the Fenton reaction, promoting peroxidation of polyunsaturated fatty acids and ultimately leading to membrane damage and cell death.^82^ Within this context, Fe/S clusters serve as critical nodes linking iron metabolism to mitochondrial function and ferroptotic vulnerability.^93^

NFS1, a core enzyme in Fe/S cluster biosynthesis, plays a pivotal role in protecting cancer cells from ferroptosis. In lung cancer cells, inhibition of NFS1 disrupts Fe/S cluster assembly and destabilizes IRP-bound Fe/S clusters, activating the iron starvation response. This response increases labile iron levels and lipid peroxidation, thereby sensitizing cells to ferroptosis. Under normoxic conditions, elevated NFS1 expression confers resistance to ferroptosis and supports tumor growth.^94^ In hypoxic triple-negative breast cancer (TNBC), targeting the CAIX-NFS1/xCT axis exacerbates mitochondrial iron accumulation, lipid peroxidation, and ferroptotic cell death through intracellular acidification, cysteine limitation, and activation of the AMPK/ACC1/ACSL4 pathway.^95^

Pharmacological and metabolic perturbations that impair Fe/S cluster integrity further highlight the link between Fe/S homeostasis and ferroptosis. Rotenone disrupts Fe/S clusters, elevates labile iron and oxidative stress, reduces GPX4 and mitochondrial complex I activity, and induces ferroptosis. Conversely, hydrogen sulfide suppresses rotenone-induced ferroptosis by enhancing NFS1-dependent Fe/S cluster biosynthesis and limiting ABCB8-mediated mitochondrial iron efflux, thereby protecting cardiomyocytes from ischemic injury.^96^ Eprenetapopt (APR-246) similarly inhibits Fe/S cluster biogenesis by suppressing NFS1 activity, depleting glutathione, amplifying ferroptosis, and restricting tumor growth, with dietary serine and glycine restriction further enhancing therapeutic efficacy.^97^

Additional Fe/S cluster proteins modulate ferroptosis sensitivity across cancer types. FXN inhibition disrupts Fe/S assembly, activates iron starvation signaling, and markedly enhances erastin-induced ferroptosis through mitochondrial iron accumulation and lipid peroxidation.^98^ In diffuse large B-cell lymphoma, silencing NFS1 or FXN suppresses iron storage proteins, enhances iron transport, promotes DNA damage, and triggers ferroptosis.^99^ Members of the CISD family further fine-tune ferroptotic responses: CISD1 (mitoNEET) limits mitochondrial iron overload and oxidative stress, whereas CISD2 loss enhances ferritin degradation, suppresses NRF2 signaling, and increases ferroptosis sensitivity in cancer cells.^100^^,^^101^ Together, these findings establish Fe/S cluster biogenesis and regulation as central determinants of ferroptosis susceptibility in cancer, revealing exploitable metabolic vulnerabilities for therapeutic intervention.

### Fe/S cluster metabolism and cuproptosis

Cuproptosis is a recently identified copper-dependent form of regulated cell death that is mechanistically linked to Fe/S cluster metabolism. Copper directly binds to lipoylated enzymes of the TCA cycle, inducing protein aggregation, loss of Fe/S proteins, and proteotoxic stress.^102^ In mammalian cells, protein lipoylation depends on the Fe/S cluster–containing enzyme LIAS, which utilizes its [4Fe–4S] cluster to catalyze sulfur insertion into fatty acyl chains. Although FDX1 is not a core component of the Fe/S assembly machinery, accumulating evidence suggests that it is functionally associated with LIAS and contributes to efficient protein lipoylation under specific conditions. Moreover, FDX1 has been implicated in the regulation of cuproptosis, positioning it as an important factor linking lipoylation status to copper-induced cell death.^103^

Copper toxicity is closely associated with Fe/S proteins and their assemblies. Excess mitochondrial Cu(I) can bind to apo-GLRX5 or ISCA1/2 heterocomplexes, preventing [2Fe-2S] cluster transfer, disrupting [4Fe-4S] maturation, and impairing Fe/S protein function.^104^ Studies on isopropylmalate dehydratase have shown that excessive copper directly attacks the Fe/S cluster, displacing iron atoms and destabilizing cluster structures.^105^ Screening of acute myeloid leukemia (AML) samples revealed UM4118, a copper ionophore that induces cuproptosis. Loss of the ISC transporter ABCB7 synergizes with UM4118, particularly in SF3B1-mutant leukemia, where mis-splicing downregulates ABCB7, thereby increasing the copper sensitivity. ABCB7 overexpression mitigates copper overload, linking Fe/S cluster defects to cuproptosis susceptibility.^106^

Cuproptosis and ferroptosis act synergistically. A dual-metallic nanozyme (HA@CuCo-NC) triggers Fe/S cluster collapse and tumoricidal ROS production. Co^2+^-induced ferroptosis causes severe mitochondrial damage and GSH depletion, which in turn activate the cuproptosis cascade. Cuproptosis suppresses Fe/S cluster formation and DLAT oligomerization, leading to Fe/S protein degradation and Fe^2+^ release into the labile iron pool. Released Fe^2+^ amplifies oxidative stress via the Fenton reaction, forming a self-reinforcing loop in which ferroptotic mitochondrial damage enhances Cu^2+^ toxicity and cuproptosis-derived Fe^2+^ further drives ferroptosis.^107^

### Fe/S clusters-linked mechanisms in other PCD

Mitochondrial Fe/S proteins modulate apoptosis by shaping mitochondrial signaling hubs. Fe/S cluster biosynthesis is impaired under hypoxia when the ISC assembly machinery is downregulated or with iron chelators. This impairs maturation of CISD2, disrupting the VDAC1 regulatory complex at the mitochondria-associated membrane and promoting VDAC1-ΔC formation with anti-apoptotic functions. This triggers mitochondrial remodeling and enhances VDAC1-ΔC interaction with Bcl-XL and hexokinase II, increasing tumor cell resistance to chemotherapy-induced apoptosis independently of Hypoxia-Inducible Factor -1α（HIF-1α) signaling.^108^ While CISD2 regulates VDAC1 cleavage-related antiapoptotic adaptation, its mechanism remains unclear. CISD2 shows antioxidant and anti-apoptotic effects through Akt/GSK-3β/Nrf2 signaling, though Fe-S cluster dependency requires investigation.^109^

In both human CIAPIN1 (anamorsin) and yeast Dre2, motif I binds a redox-active [2Fe-2S] cluster that accepts electrons from NDOR1/Tah18 to support CIA-dependent processes. In contrast, motif II shows species-specific cluster coordination. Both human anamorsin and its yeast homolog Dre2 are capable of coordinating an additional Fe/S cluster at this site; human anamorsin predominantly binds a second [2Fe-2S] cluster with unusual electronic properties, whereas yeast Dre2 has been shown by EPR and mutational analyses to coordinate an oxygen-sensitive [4F-4S] cluster.^110^ Beyond its role in Fe/S metabolism, the Tah18-Dre2 complex in yeast responds to oxidative stress and triggers mitochondria-mediated apoptosis via Tah18-dependent nitric oxide production,^111^ a function that is conserved in the human anamorsin/NDOR1 system. Anamorsin was originally identified as an anti-apoptotic protein and was later shown to be a direct substrate of caspase-3, whose cleavage abolishes its cytoprotective activity and promotes neuronal death.^112^ In cancer and proliferative diseases, CIAPIN1 is frequently upregulated and enhances cell survival, proliferation, migration, and metabolic reprogramming through the activation of PI3K/Akt and JAK2/STAT3 signaling while suppressing oxidative stress and apoptosis.^113^^,^^114^ However, whether CIAPIN1-mediated Fe/S cluster assembly is mechanistically linked to its regulation of apoptosis remains unclear.

PANoptosis is a unique form of inflammatory cell death integrating pyroptosis, apoptosis, and necroptosis via the PANoptosome complex.^115^ In CRC, NFS1 is transcriptionally upregulated by MYC and correlates with poor prognosis and chemotherapy resistance. Oxaliplatin-induced oxidative stress promotes phosphorylation of NFS1 at Ser293, which inhibits PANoptosis in a phosphorylation-dependent manner. Conversely, selective inhibition or loss of NFS1 in CRC cells disrupts Fe/S cluster homeostasis, increases ROS, and cooperates with oxaliplatin to trigger PANoptosis, thereby enhancing chemosensitivity.^116^ These findings indicate that, although NFS1 is essential for normal cell function, its tumor-specific overexpression and context-dependent inhibition represent a potential strategy to improve the efficacy of platinum-based chemotherapy by targeting Fe/S cluster biosynthesis.

## Abnormal Fe/S cluster assembly and related diseases

Defects in Fe/S cluster biogenesis, trafficking, and utilization are associated with a broad spectrum of human diseases, reflecting the central roles of Fe/S proteins in mitochondrial respiration, iron homeostasis, and genome maintenance. Pathogenic variants in genes involved in mitochondrial Fe/S cluster assembly, including FXN, ISCU, FDX2, LYRM4, NFU1, BOLA3, ISCA1/2, and IBA57, are primarily linked to neuromuscular and metabolic disorders, such as neurodegeneration, myopathy, lactic acidosis, and severe mitochondrial dysfunction syndromes. These phenotypes are generally attributed to impaired oxidative phosphorylation, defective Fe/S-dependent enzyme activity, and mitochondrial iron misdistribution.

In addition, Fe/S cluster defects affecting respiratory chain components (e.g., NDUFS1/2, SDHB, and UQCRFS1) compromise electron transport and cellular energy metabolism, further underscoring the dependence of mitochondrial function on intact Fe/S biology. Beyond mitochondria, numerous cytosolic and nuclear Fe/S proteins—including DPD, DPH1/2, CIAO1, MMS19, MUTYH, XPD, FANCJ, DDX11, RTEL1, POLD1, and POLE—play essential roles in DNA replication, repair, and translation. Dysfunctions in these proteins have been associated with developmental abnormalities, genome instability syndromes, premature aging phenotypes, and increased susceptibility to malignancy, although cancer development is not an obligatory outcome of Fe/S pathway impairment.

Perturbations in Fe/S cluster trafficking and iron handling, such as defects in ABCB7 or GLRX5, result in sideroblastic anemia and mitochondrial iron overload, highlighting the tight coupling between Fe/S metabolism and cellular iron homeostasis. Importantly, while many Fe/S-related disorders are not classified as cancers, they affect biological processes—such as redox balance, DNA integrity, and metabolic flexibility—that are frequently rewired in tumor cells. Thus, Fe/S cluster dysregulation provides a mechanistic framework linking multisystem disease phenotypes with pathways that are also relevant to tumor biology, rather than establishing a direct or universal causal relationship with cancer (see Tables 1 and 2 for details).

**Table 1:** Associated disease related to Fe/S clusters biogenesis

Protein	Main function	Associated disease	Reference
**ISC assembly factors involved in *de novo* Fe/S cluster synthesis**			

ISCU2	scaffold protein	hereditary myopathy with lactic acidosis (HML)	Olsson et al.^117^
NFS1	cysteine desulfurase	combined oxidative phosphorylation deficiency 52 (COXPD52)	Farhan et al.^118^
ISD11	NFS1 stabilizer	combined oxidative phosphorylation deficiency 19 (COXPD19)	Lim et al.^119^
FXN	allosteric NFS1 activator	Friedreich ataxia (FRDA)	Rötig et al.^120^
FDX 2	electron transfer	paroxysmal mitochondrial myopathy with optic atrophy and reversible leukoencephalopathy (MEOAL)	Gurgel-Giannetti et al.^121^

**ISC factors involved in cluster transfer**			

HSPA9	Hsp70 chaperone	anemia, sideroblastic, 4(SIDBA4)	Schmitz-Abe et al.^122^
HSC20	co-chaperone	anemia, sideroblastic, 5(SIDBA5)^a^	Crispin et al.^123^
GLRX5	[2Fe-2S] transfer	anemia, sideroblastic, 3, pyridoxine-refractory (SIDBA3)	Liu et al.^124^

**Late-acting ISC factors involved in assembly and insertion of Fe/S clusters into target proteins**			

ISCA1	[4Fe-4S] carrier	multiple mitochondrial dysfunctions syndrome 5(MMSD5)	Shukla et al.^125^
ISCA2	[4Fe-4S] carrier	multiple mitochondrial dysfunctions syndrome 4(MMSD4)	Alaimo et al.^126^
IBA57	assembly factor	multiple mitochondrial dysfunctions syndrome 3(MMSD3)	Ajit Bolar et al.^127^
NFU1	targeting factor	multiple mitochondrial dysfunctions syndrome 1(MMSD1)	Wachnowsky et al.^128^
BOLA3	targeting co-factor	multiple mitochondrial dysfunctions syndrome 2 (MMSD2)	Cameron et al.^129^
NUBPL	complex I targeting	mitochondrial complex I deficiency, nuclear type 21	Protasoni et al.^130^

**(Fe-S) int transfer to cytoplasm**			

ABCB7	(Fe-S) int exporter	X-linked sideroblastic anemia with ataxia (XLSA/A)	Boultwood et al.^131^

**Components of the CIA machinery**			

NUBP1	cytosolic scaffold	depression	Shukla et al.^125^
NUBP2	cytosolic scaffold	oral squamous cell carcinoma	Abend et al.^132^
NDOR1	electron transfer	thyroid cancer	He et al.^133^
CIAPIN1	[2Fe-2S] carrier	NA	NA
GLRX3	[2Fe-2S] auxiliary chaperone	nasopharyngeal carcinoma	Bost et al.^134^
CIAO3	4Fe-4S] cluster transfer	alcohol addiction	Reiss et al.^135^
CIAO1	targeting hub	neuromuscular disease	Maio et al.^136^
MMS19	targeting adapter	neurodegenerative disease	van Karnebeek et al.^137^

CIA, cytosolic iron-sulfur assembly; ISC, mitochondrial Fe/S clusters assembly.

a Denotes a tentative relationship between genotype and phenotype.

**Table 2:** Diseases associated with nuclear Fe/S proteins

Protein	Primary function	Disorder	Reference
POLD1	DNA replication (lagging strand synthesis) and DNA repair (BER/NER)	colorectal cancer, susceptibility to, 10^a^	Roberts et al.^138^
		mandibular dysplasia, hearing loss, progeroid features, and fat dysplasia (MDPL)	Weedon et al.^139^
POLE	DNA replication (leading strand synthesis) and DNA repair (NER)	colorectal cancer, susceptibility to, 12^a^	Tomas-Roca et al.^140^
		FILS syndrome	Ronchi et al.^141^
		IMAGE syndrome	Logan et al.^142^
REV3L	translesion synthesis (TLS) and DNA damage tolerance	moebius syndrome	Di Lazzaro Filho et al.^143^
DNA2	okazaki fragment processing and double-strand break repair (resection)	progressive external ophthalmoplegia with mitochondrial DNA deletions, autosomal dominant 6	Shaheen et al.^144^
		Rothmund-Thomson syndrome, type 4	Boulouard et al.^145^
		Seckel syndrome 8	De Nicolo et al.^146^
MUTYH	BER (A:8-oxoG glycosylase)	MUTYH-associated polyposis (MAP)	D’Agostino et al.^147^
NTHL1	BER (oxidized pyrimidine glycosylase)	familial adenomatous polyposis 3	Kamal et al.^148^
FANCJ	interstrand crosslink repair (Fanconi anemia pathway) and G-quadruplex resolution	breast cancer, early onset, susceptibility to	Willemsen et al.^149^
		Fanconi anemia, complementation group J	Gowans et al.^150^
DDX11	sister chromatid cohesion	Warsaw breakage syndrome (WABS)	Alkhunaizi et al.^151^
KIF4A	chromosome condensation and DNA repair (PARP1 interaction)	intellectual developmental disorder, X-linked 100	Lehmann^152^
		taurodontism, microdontia, and dens invaginatus	Graham et al.^153^
XPD	NER (TFIIH subunit)	xeroderma pigmentosum (XP)	Rudolf et al.^154^
		trichothiodystrophy (TTD)	Tang et al.^155^
		cerebrooculo-facio-skeletal syndrome 2^b^	Mukhopadhyay et al.^156^

BER, base excision repair; NER, nucleotide excision repair.

a Numbers represent the identified distinct germline susceptibility loci for colorectal cancer.

b Denotes a tentative relationship between genotype and phenotype.

## Roles and therapeutic targeting of Fe/S proteins in cancer

Dysregulation of Fe/S cluster biology not only precipitates metabolic collapse and PCD but also significantly influences mitochondrial respiration, DNA repair, and redox homeostasis. This makes it a promising biomarker for cancer progression and therapeutic responses (Table 3). Recent research has led to the identification of small-molecule inhibitors, metal-based compounds, and nanotechnology-based delivery systems, all of which can regulate Fe/S cluster assembly and Fe/S protein function with remarkable selectivity. These strategies exploit the metabolic vulnerabilities of cancer cells while precisely modulating Fe/S cluster-dependent pathways.

**Table 3:** Physiological functions of Fe/S proteins and associated metabolic regulators in cancer

Types of cancer	Fe/S Network targets	Effect on cancer	Mechanisms	Reference
Renal cancer	NFU1, ISCA1/2	inhibit	high expression means a better prognosis	Yang et al.^157^
ccRCC	*ISCU*	inhibit	VHL-HIF-miR-210 axis suppresses *ISCU*/metabolic shift to anaerobic respiration	Neal et al.^158^
HLRCC	ACO2	inhibit	fumarate accumulation causes succination of Fe/S cluster binding cysteines/impairs enzyme activity	Ternette et al.^62^
RCC	ACO2	inhibit	metabolic adaptation/immune evasion	Jaworski et al.^159^
PDAC	NCOA4	promote	ferritinophagy/iron maintenance for Fe/S cluster synthesis	Santana-Codina et al.^160^
CRC	NFS1	promote	prevent PANoptosis/reduce ROS	Lin et al.^116^
	MUTYH	inhibit	Fe-S cluster required for DNA binding/DNA repair (BER)	Brinkmeyer and David et al.^161^
HNSC	ISCA2	promote	suppress CD4^+^ T cell activation/antigen presentation	Zheng et al.^162^
LUAD	ABCE1	promote	interact with β-actin/regulate cytoskeletal polymerization	Yu et al.^163^
NSCLC	CISD2 (NAF-1)	promote	maintain mitochondrial function/prevent ROS accumulation/iron homeostasis	Shao et al.^164^
	ACO2	inhibit	regulate iron homeostasis/increase labile iron pool	Mirhadi et al.^165^
	NDUFS2	promote	upregulated by S100A4 to promote OXPHOS	Liu et al.^166^
HCC	CISD2	promote	NRAV/miR-199a-3p/CISD2 axis activates Wnt/β-catenin signaling	Wang et al.^167^
Liver cancer	*ISCU*	inhibit	p53-*ISCU* axis regulates iron homeostasis/modulate IRP1-FTH1 interaction	Funauchi et al.^168^
Breast cancer	NDUFS3	inhibit	regulate ROS-mediated metabolic switch/maintain OXPHOS	Wang et al.^169^
	SDHB and NDUFS3	inhibit	bioenergetic alterations/downregulation of OXPHOS complexes	Putignani et al.^170^
	NAF-1 (CISD2)	promote	regulate Fe distribution/mitochondrial metabolism/HIF1α pathway	Holt et al.^171^
	mitoNEET and NAF-1	promote	prevent iron overload/support glycolysis	Bai et al.^172^
Oral cancer	NDUFS8	promote	upregulate NDUFS8 to increase ROS/activate MAPK & Ras-ERK signaling	Cheng et al.^173^
Glioblastoma multiforme (GBM)	complex I	promote	drive reverse electron transfer (RET)/regulate NAD+/NADH balance	Ojha et al.^174^
Breast and ovarian cancer	FANCJ (BRIP1)	inhibit	Fe-S cluster required for G-quadruplex (G4) unwinding/DNA repair	Odermatt et al.^175^

ETC, electron transport chain; OXPHOS, oxidative phosphorylation; ccRCC, clear cell renal cell carcinoma; HLRCC, hereditary leiomyomatosis and renal cell cancer; NSCLC, non-small cell lung cancer; PDAC, pancreatic ductal adenocarcinoma; HNSC, head and neck squamous cell carcinoma; LUAD, lung adenocarcinoma; HCC, hepatocellular carcinoma; OCCC, ovarian clear cell carcinoma; CRC, colorectal cancer.

### Fe/S cluster metabolism as a central regulator of tumor cell fitness

Here, tumor cell fitness is defined as the systems-level ability of cancer cells to coordinate metabolic adaptation, genome maintenance, and stress tolerance, processes that are critically supported by Fe-S protein function. Fe/S proteins play a bidirectional regulatory role in tumorigenesis and development. Studies show that in clear cell renal cell carcinoma (KIRC), mitochondrial Fe-S proteins such as ISCA1, ISCA2, IBA57, and NFU1 are expressed at lower levels than in normal tissue, while high transcription levels significantly associate with overall survival (OS) and disease-free survival (DFS), suggesting their tumor-suppressive role in metabolic homeostasis.^157^ In head and neck squamous cell carcinoma (HNSC), cuproptosis-related genes (CRGs)—including Fe-S cluster biosynthesis proteins (ISCA2, GLRX5, NDUFA1, and NDUFB2)—are upregulated and linked to poor prognosis, promoting tumor progression through metabolic reprogramming, cell cycle progression, and immune suppression.^162^ In ccRCC, VHL loss leads to HIF accumulation and hypoxia adaptation, while upregulating miR-210, which suppresses ISCU1/2, weakening mitochondrial Fe/S-dependent respiratory chain function and promoting tumor proliferation.^158^ Low ACO2 expression in RCC and non-small cell lung cancer (NSCLC) associates with poor prognosis, as its loss impairs TCA cycle flux and oxidative metabolism, making cancer cells rely on glycolysis.^159^^,^^176^ CISD2, highly expressed in NSCLC and cervical cancer, regulates iron homeostasis through aconitase activity while protecting mitochondrial function. Its knockdown leads to iron accumulation and mitochondrial damage, inhibiting cancer cell proliferation.^164^ The Fe/S DNA helicase BRIP1 contains a [4Fe-4S] cluster maintaining DNA repair functions. Its upregulation in multiple cancers promotes cell survival through DNA damage repair and is linked to genomic instability, serving as a pan-cancer biomarker.^177^ Fe/S proteins' effects depend on their roles in metabolism, iron homeostasis, and DNA repair: their loss can promote tumors by disrupting stability, while excessive activity can enhance tumor proliferation.

The biosynthesis of Fe/S cluster is not only a fundamental component of cellular metabolism, but also a key defense mechanism that tumor cells use to cope with environmental stress and chemotherapy induced damage. Disruption of ISC assembly can also significantly affect tumor treatment responses by weakening DNA repair capacity. Sideroflexin 4(SFXN4) is upregulated in ovarian cancer; its knockdown inhibits Fe/S cluster biosynthesis, which on one hand leads to abnormal mitochondrial iron accumulation and triggers oxidative stress and DNA damage, and on the other hand impairs the function of multiple Fe/S-dependent DNA repair proteins (including FANCJ, XPD, and DNA polymerase δ), thereby simultaneously compromising the homologous recombination repair (HRR) and NER pathways. This markedly increases the sensitivity of ovarian cancer cells to cisplatin and PARP inhibitors, suggesting a close relationship between Fe/S cluster assembly status and chemotherapeutic response.^178^ Similarly, in cisplatin-resistant NSCLC, tumor cells rely on oxidative phosphorylation centered on the Fe-S protein SDHB to maintain mitochondrial function and survival under glutamine deprivation. The iron chelator Deferoxamine (DFO) can activate the c-Jun N-terminal Kinase (JNK) signaling pathway to induce autophagic cell death and apoptosis, thereby effectively suppressing the growth of cisplatin-resistant NSCLC cells.^179^

The CIA pathway supports DNA replication and repair by maintaining Fe/S protein maturation. Its functional deficiency does not generally increase DNA damage sensitivity, but instead selectively weakens tumor cells' ability to tolerate replication stress. TNBC cells lacking key components of the CIA targeting complex—MMS19 or CIA2OB—show significantly greater sensitivity to ATR or Chk1 inhibitors than to cisplatin, PARP inhibitors, or hydroxyurea. When ATR or Chk1 is inhibited, abnormal activation of dormant replication origins greatly increases replication fork density, thereby sharply increasing the need for DNA primases and Fe/S-dependent DNA polymerases. This makes cells with impaired CIA function more prone to replication catastrophe. Additionally, impairment of the CIA pathway disrupts nucleotide homeostasis, further reducing replication fidelity and exacerbating genomic instability.^180^

Abnormal regulation of MMS19 is also associated with drug resistance phenotypes. For example, in bladder cancer, c-MYC directly transcriptionally activates MMS19, enhancing tumor cell resistance to cisplatin and promoting tumor progression, although the precise biochemical mechanisms have yet to be fully elucidated.^181^ In glioblastoma, glioma stem cells (GSCs) relieve inhibition of the CIA pathway centered on MMS19 through PTEN succinylation, thereby reactivating CIA, promoting GSC maintenance and tumorigenesis. Further studies reveal that this modification stems from the highly active *de novo* purine synthesis pathway in GSCs; the key enzyme, adenylosuccinate lyase (ADSL), produces fumarate, which promotes succinylation of PTEN at C211. Re-expression of PTEN C211S or treatment with NAC can significantly enhance the sensitivity of GSC-derived tumors to temozolomide (TMZ) and radiotherapy by weakening DNA damage repair mediated by the CIA pathway, thereby prolonging survival in mouse models.^182^

At the level of iron homeostasis regulation, expression of *ISCU* in hepatocellular carcinoma tissues is reduced in most samples and is significantly associated with p53 mutation. Wild-type p53 can directly induce *ISCU* transcription, thereby maintaining the balance of iron storage and uptake via regulation of the IRP-IRE system. Conversely, loss of p53 function causes *ISCU* downregulation, iron homeostasis imbalance, and iron overload under DNA damage conditions, revealing a previously unrecognized function of p53 in maintaining iron homeostasis and suppressing hepatocellular carcinoma–related iron overload by regulating Fe/S cluster biogenesis. However, the role of this pathway in systemic iron homeostasis and therapeutic response requires further clarification.^168^ In addition, while the clinical drug eprenetapopt was initially developed as a mutant p53 reactivator, its primary antitumor effect derives from depleting GSH and inhibiting NFS1-mediated Fe/S cluster biogenesis, thereby disrupting redox homeostasis and inducing ferroptosis. This process is independent of p53 status and is highly dependent on iron and cysteine metabolism, further highlighting the potential value of targeting the Fe/S cluster pathway in cancer therapy.^183^

Collectively, Fe/S cluster metabolism functions as a central metabolic hub that integrates oncogenic signaling with mitochondrial homeostasis, enabling cancer cells to adapt dynamically to metabolic and environmental fluctuations.

### Small-molecule strategies targeting Fe/S proteins and their assembly machinery

**Figure 4:**
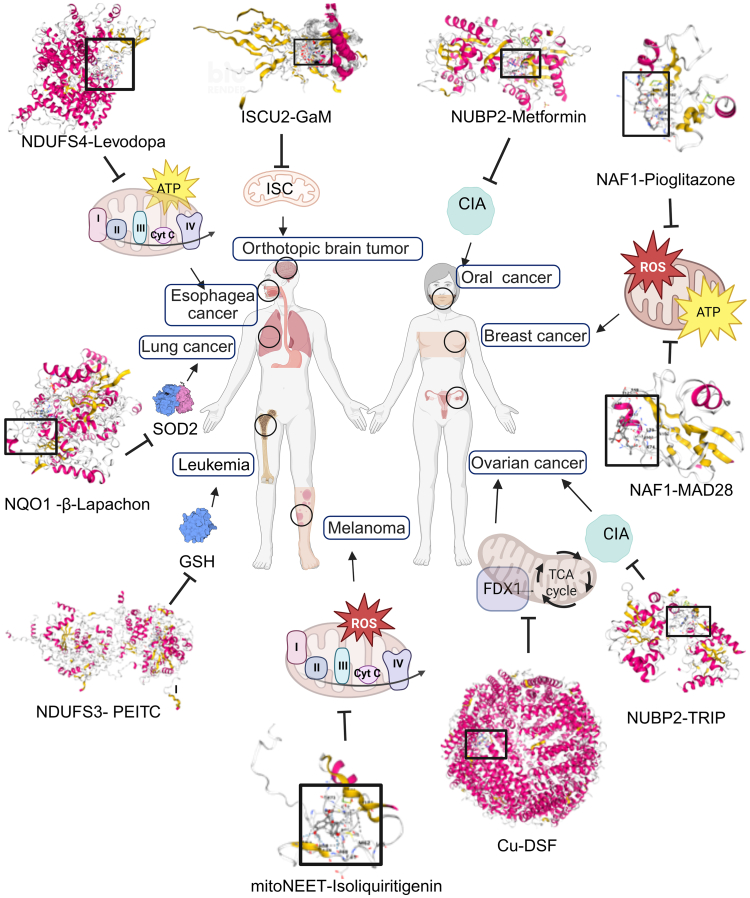
Therapeutic strategies targeting Fe/S protein for cancers with small molecule modulators The inhibition of Fe/S protein and their biosynthetic pathways could be an effective anticancer strategy. Drugs or small-molecule modulators that bind to Fe/S clusters biosynthesis proteins or ISC-related proteins can inhibit cancer progression by suppressing Fe/S clusters synthesis and modulating oxidative stress, energy metabolism, and mitochondrial dysfunction, thus emerging as new targets for cancer therapy. Molecular docking demonstrated the binding of these drugs and small-molecule modulators to their molecular targets. DSF, disulfiram; GaM, gallium maltolate; PEITC, β-phenethyl isothiocyanate; TRIP, rhenium tricarbonyl compound. The image was created in https://BioRender.com.

Current research indicates that inhibiting Fe/S cluster and their biosynthetic pathways may be an effective anti-cancer strategy. Some of these approaches have already entered clinical trials. The following section introduces several drugs and small-molecule modulators (Table 4) and their mechanisms of targeting Fe/S cluster (Figure 4).

**Table 4:** Therapeutic strategies targeting Fe/S cluster metabolism and associated proteins in cancer

Types of cancer	Drugs and small molecule modulators	Chemical structure	Targets	Phase	ClinicalTrial. gov ID	Reference
Orthotopic brain tumor	GaM		ISCU2	1	NCT04319276	Alhajala et al.^184^
Oral squamous cell carcinoma	metformin		NUBP2	2	NCT03076281	Ji et al.^185^
Ovarian cancer	TRIP		NUBP2	NA	NA	Neuditschko et al.^186^
Breast cancer	pioglitazone		NAF-1	2B	NCT05013255	Darash-Yahana et al.^187^
Breast cancer	MAD-28		NAF-1	NA	NA	Bai et al.^172^
Ovarian cancer	DSF		Cu	NA	NA	Gan et al.^188^
Melanoma	ISL		mitoNEET	NA	NA	Chen et al.^189^
Esophageal squamous cell carcinoma	levodopa		NDUFS4	NA	NA	Li et al.^190^
Lung cancer	β-lapachon		NQO1	NA	NA	Jiang et al.^191^
Leukemia	PEITC		NDUFS3	NA	NA	Chen et al.^192^

DSF, disulfiram, GaM, gallium maltolate; PEITC, β-phenethyl isothiocyanate; TRIP, rhenium tricarbonyl compound; ISL, isoliquiritigenin.

In CRC, NFS1 deletion reduces oxaliplatin resistance, and NUBP2 knockdown decreases cell proliferation and promotes apoptosis. Additionally, gallium maltolate (GaM) binds to ISCU2, inhibiting Fe/S cluster assembly and enhancing cytotoxicity in glioblastoma cells.^184^ ISCA2 inhibitors significantly reduce tumor growth in clear cell renal cell carcinoma by blocking IRE-dependent translation.^193^ Further studies have identified NUBP2 and Fe/S cluster biosynthetic pathways as potential therapeutic targets in cancer treatment. Metformin inhibits the proliferation and migration of oral squamous cell carcinoma by regulating NUBP2 alternative splicing. As a key protein in Fe/S cluster assembly, alterations in NUBP2 splicing affect Fe/S cluster synthesis and suppress cancer cell growth.^185^ In ovarian cancer cells, the rhenium tricarbonyl compound (TRIP) competes with NUBP2 for binding, disrupting Fe/S cluster biogenesis, leading to the downregulation of Fe/S proteins, and upregulating ferritin as a feedback response to Fe/S cluster depletion.^186^ Overexpression of NAF-1 correlates with larger breast tumors, whereas stabilizing NAF-1 clusters through mutations or pharmacological interventions inhibits breast cancer cell growth.^187^ Moreover, MAD-28, a specially designed compound, destabilized the [2Fe-2S] clusters in NAF-1 and the mitoNEET. By disrupting the bond between histidine ligands and cluster iron, MAD-28 reduces mitochondrial respiration and increases glycolysis in breast cancer cells without affecting normal breast cells. Isoliquiritigenin (ISL), derived from the root of Glycyrrhiza glabra L., reduces mitoNEET expression in melanoma cells, increases ROS levels, and induces mitochondrial dysfunction, ultimately promoting melanoma cell death.^157^ Disulfiram (DSF), a traditional anti-alcoholism drug, has shown potential anticancer effects. DSF downregulates FDX1 and Fe/S proteins by binding to copper ions, inhibiting ovarian cancer cell viability, reducing tumor volume, and improving survival in ovarian cancer xenograft models.^188^

### Fe/S cluster destabilization-driven nanotherapy

Although Fe/S proteins play a crucial role in tumor metabolism, directly targeting them for therapy remains challenging due to their intracellular localization and lack of conventional drug-binding sites. Systemic administration of metal ions or redox-active agents that disrupt Fe/S cluster is often limited by non-specific toxicity. Nanoparticle-based therapeutic strategies offer solutions to these limitations. By enabling controlled delivery and tumor microenvironment-responsive activation, nanomaterials can selectively disrupt Fe/S protein homeostasis within cancer cells while minimizing off-target effects. Against this backdrop, a series of emerging nanotherapeutic strategies have been designed around the initial event of “Fe/S clusters destabilization,” thereby transforming mitochondrial metabolic vulnerability into a self-propagating cascade of cell death dominated by cuproptosis and its crosstalk with ferroptosis.

A representative example is the hyaluronic acid (HA)-functionalized Cu/Co bimetallic nanozyme (HA@CuCo-NC), which was designed to hijack Fe/S clusters metabolism and trigger a self-amplifying “cuproptosis-ferroptosis” loop in osteosarcoma. Tumor-specific accumulation achieved via HA-CD44 recognition induces excessive oxidative stress and GSH depletion, leading to impaired biosynthesis of Fe/S clusters and degradation of key Fe/S proteins such as FDX1 and LIAS. Meanwhile, Cu2+ promotes abnormal oligomerization of lipoylated DLAT (a molecular marker of cuproptosis), thus accelerating Fe/S protein destabilization and mitochondrial metabolic collapse. Importantly, the breakdown of Fe/S clusters releases Fe2+ into the labile iron pool, further amplifying lipid peroxidation through Fenton chemistry and activating ferroptosis. The mutual reinforcement among Fe/S disruption, cuproptosis, and ferroptosis establishes a closed-loop death cascade, making Fe/S clusters a convergence point in bimetallic nanozyme anti-cancer strategies.^194^

Consistent with this Fe/S clusters-centered framework, bimetallic nanostructures have been shown to exploit TNBC intrinsic dependence on copper and iron. CuFeTe2 nanosheets release Cu+ and Fe2+ in the acidic tumor microenvironment, where Cu+ induces DLAT aggregation and destabilization of mitochondrial Fe/S proteins to trigger cuproptosis, while Fe2+-driven redox imbalance simultaneously amplifies ferroptosis. Near-infrared-II (NIR-II) photothermal activation further lowers the stability threshold of Fe/S clusters, intensifies oxidative stress, and enhances the synergistic cytotoxicity of these two cell death pathways.^195^ Similarly, CaO2@CuPDA nanomedicine amplifies cuproptosis by promoting the Cu2+/Cu+ redox cycle and H2O2 production, where FDX1-dependent Cu2+ reduction accelerates the destruction of Fe/S clusters and propagates Fe/S clusters-dependent copper toxicity, a process further enhanced under Ca2+ overload and photothermal effects.^196^

In addition to directly delivering metals, some nanoplatforms also exploit tumor-specific redox remodeling to enhance the vulnerability of Fe/S clusters. Prodrug-based copper-chelating nanoparticles (PCD@Cu) and biomimetic NIR-II cuproptosis amplifiers (PCD@CM) selectively increase intracellular copper retention via the copper transporter ATPase 2 (ATP7B), thereby promoting Cu+ accumulation, lipoacylated DLAT aggregation, and the subsequent depletion of mitochondrial iron-sulfur proteins such as FDX1 and LIAS. In these systems, the loss of Fe/S protein is not a bystander effect but is a decisive factor in copper-induced proteotoxic stress and immunogenic cell death.^197^^,^^198^ Similarly, copper-gallic acid metal-phenolic network nanoparticles (Cu-GA NPs) and photoactivated copper ion carriers (CJS-Cu NPs) utilize tumor-enriched glutathione and spatiotemporally controllable copper redox conversion to induce targeted Fe/S clusters damage, thereby conferring spatial precision to the cuproptosis induction process.^199^^,^^200^

In summary, these studies depict a unified “Fe/S clusters collapse-driven” paradigm for nanotherapy: engineered nanomaterials not only deliver cytotoxic metals but also strategically disrupt Fe/S clusters homeostasis to reshape mitochondrial metabolism, enhance copper dependency, and couple cuproptosis with ferroptosis. By placing iron-sulfur clusters at the forefront of metal-induced cell death signaling, this framework provides a mechanistic basis for developing next-generation nanotherapies that transform metabolic vulnerabilities into programmable anti-cancer strategies.

## Conclusion and future perspectives

Studies have shown that disruption of Fe/S clusters biogenesis alters mitochondrial respiration, DNA repair, and redox signaling, thereby promoting tumorigenesis and therapy resistance. Excessive Fe/S clusters depletion can induce metabolic collapse and trigger PCD, positioning Fe/S clusters homeostasis as a key determinant of cancer progression. Future research should examine how cancer cells remodel Fe/S clusters metabolism in response to stress. The molecular crosstalk between the Fe/S clusters biosynthetic machinery and oncogenic regulators, such as MYC, NRF2, and p53, remains poorly understood. Understanding this network is essential for identifying therapeutic vulnerabilities. Another avenue lies in studying the non-canonical roles of Fe/S proteins beyond electron transfer, such as RNA modification and immune evasion, which may expand druggable Fe/S clusters targets.

From a translational perspective, the development of agents that selectively destabilize or inhibit Fe/S clusters assembly, such as DSF-copper complexes or NAF-1 inhibitors, offers a novel strategy for impairing cancer metabolism. Nanoparticle-based platforms can further enhance specificity by delivering Fe/S clusters-targeting compounds directly to the tumor mitochondria, minimizing systemic toxicity. The integration of Fe/S clusters-targeted therapy with ferroptosis or DNA damage-inducing agents may also yield synergistic antitumor effects.

A major challenge lies in establishing reliable biomarkers that reflect Fe/S clusters dysfunction and predict therapeutic response. Advances in redox proteomics, imaging, and metabolic flux analysis may soon enable the dynamic monitoring of Fe/S clusters status in tumors. Ultimately, decoding the regulatory networks that govern Fe/S clusters biology will not only deepen our understanding of cancer metabolism, but also unveil a new generation of metabolic therapies that exploit the intrinsic fragility of the Fe/S clusters system.

## Acknowledgments

This work was supported by grants from the 10.13039/501100001809National Natural Science Foundation of China (nos. 82571958 and 82571959) and the Supporting China Medical University’s High-Quality Development Science and Technology Fund Project of Liaoning Province (2023JH2/20200131).

## Author contributions

S.Y. and H.G., investigation, writing – original draft, writing – review, and editing; J.C. and X.L., conceptualization, funding acquisition, writing, review, and editing. All the authors have read and approved the final manuscript.

## Declaration of interests

The authors declare no conflicts of interest.
